# Enteropathy in inborn and acquired errors of immunity: histological patterns and clinical correlates

**DOI:** 10.3389/fimmu.2026.1934715

**Published:** 2026-10-15

**Authors:** Meera Thangarajah, Samar Ojaimi, Alisa Kane, Sanjay Swaminathan, Ming Wei Lin, Alberto Pinzon-Charry, Tiarni Templeton, Peter McNaughton, Thanh-Thao Adriana Le, Constance H. Katelaris, Julian Bosco, Lucinda J. Berglund

**Affiliations:** 1 Faculty of medicine and health, University of Sydney, Sydney, NSW, Australia; 2 Immunology Department, Westmead Hospital, Sydney, NSW, Australia; 3 Centre for Immunology and Allergy Research, Westmead Institute of Medical Research, Sydney, NSW, Australia; 4 Clinical Immunogenomics Research Consortium Australasia (CIRCA), Sydney, NSW, Australia; 5 Immunology and Allergy Unit, Monash Health, Melbourne, VIC, Australia; 6 Monash University, Melbourne, VIC, Australia; 7 SydPath, Sydney, NSW, Australia; 8 The Garvan Institute of Medical Research, Sydney, NSW, Australia; 9 University of New South Wales, Sydney, NSW, Australia; 10 Immunology Department, St Vincent’s Hospital, Sydney, NSW, Australia; 11 Immunology Department, Blacktown Hospital, Sydney, NSW, Australia; 12 Western Sydney University, Sydney, NSW, Australia; 13 Institute of Clinical Pathology and Medical Research, Sydney, NSW, Australia; 14 Immunology Department, Queensland Children’s Hospital, Queensland, QLD, Australia; 15 University of Queensland, Queensland, QLD, Australia; 16 Griffith University, Queensland, QLD, Australia; 17 Immunology Department, Royal Hobart Hospital, Hobart, TAS, Australia; 18 Murdoch Children’s Research Institute, Melbourne, VIC, Australia; 19 Immunology Department, Campbelltown Hospital, Sydney, NSW, Australia; 20 Immunology Department, Alfred Health, Melbourne, VIC, Australia; 21 The Jeffrey Modell Diagnostic and Research Centre for Primary Immunodeficiencies, Melbourne, VIC, Australia

**Keywords:** common variable immunodeficiency, enteropathy, gastrointestinal disease, histopathology, immune dysregulation, inborn errors of immunity

## Abstract

**Introduction:**

Enteropathy is a significant and under-recognised complication of inborn errors of immunity (IEI), frequently misclassified as inflammatory bowel disease (IBD) or coeliac disease due to overlapping and non-specific histopathological findings. Diagnostic criteria remain poorly defined, contributing to substantial diagnostic delay and associated morbidity.

**Methods:**

We conducted a structured literature review and established the AT-SEA (Adult Tertiary Severe Enteropathy Audit) working group, a collaborative network of Australian clinical immunologists operating under the Clinical Immunogenomics Research Consortium Australasia (CIRCA), to characterise the clinicopathological features of IEI-associated enteropathy across a multicentre cohort.

**Results:**

Twenty-eight patients were identified. Moderate or severe disease was present in 89%, with malabsorption in 71% and protein-losing enteropathy in 43%. Across 48 biopsy reports, the most frequent histopathological patterns were coeliac-like changes (including villous atrophy, increased intraepithelial lymphocytes and crypt hyperplasia), IBD-like changes, reduced or absent plasma cells, graft versus host disease (GVHD)-like changes or increased apoptotic bodies and intraepithelial neutrophilia. Seventy-seven percent of patients demonstrated two or more concurrent patterns. Increasing histological complexity correlated significantly with clinician-assessed disease severity (Kruskal-Wallis p=0.042).

**Discussion:**

IEI-associated enteropathy is characterised by overlapping and evolving histopathological patterns rather than discrete subtypes. The co-occurrence of multiple patterns should prompt consideration of an underlying IEI.

## 1 Introduction

Inborn errors of immunity (IEI) encompass a rapidly expanding group of over 500 disorders characterised by immune deficiency, immune dysregulation, or both, spanning humoral, cellular, combined, and innate immune defects. In adults, an IEI-like phenotype may arise without an identified pathogenic germline variant, termed Acquired Errors of Immunity (AEI), including phenocopies due to somatic variants and conditions clinically and immunologically defined as common variable immunodeficiency (CVID) in whom a causative mutation has not been found (1). In routine practice, the distinction between IEI and AEI is not always clear, hence we use the term IEI/AEI where appropriate in this article. Gastrointestinal manifestations are evident in 5-50% of patients with primary immunodeficiency disorders and are highly variable. They can be further categorised into infectious, malignant, inflammatory and autoimmune phenomena (2–5). Of these, “enteropathy” is a commonly identified non-infectious complication of IEI/AEI which can cause debilitating sequelae such as malabsorption and frank malnutrition (5). A national immunodeficiency registry analysis identified that patients with enteropathy had significantly lower Karnofsky-Lansky functional scores than those without, and a four-fold increased mortality risk (6, 7). Despite its impact, IEI/AEI-associated enteropathy remains poorly characterised with a limited understanding of its pathophysiology and a lack of clear diagnostic criteria, leading to delays in disease-specific treatment and contributing to significant morbidity and mortality.

Enteropathy can be the initial presenting manifestation of an IEI/AEI, including in adults and in patients without a known family history of IEI/AEI (8, 9). IEI/AEI-associated enteropathy often presents with chronic diarrhoea, bloating and weight loss, while abdominal pain, fever and haematochezia have also been reported (10, 11). Intestinal biopsies from patients with IEI/AEI-associated enteropathy demonstrate an array of histopathological abnormalities that are often non-specific or mimic well-known gastrointestinal (GI) diseases, especially Coeliac Disease (CD) and IBD (6, 12–14). Recurring mucosal patterns described across published case series include villous atrophy, increased intraepithelial lymphocytosis (IEL), reduced or absent lamina propria plasma cells, nodular lymphoid hyperplasia (NLH), GVHD-like epithelial injury with crypt apoptosis, and IBD-like crypt-destructive inflammation, often coexisting within the same patient (10, 15, 16). In the absence of consensus diagnostic criteria, patients are frequently misclassified as having refractory CD or IBD and managed accordingly for months to years before an underlying immunological diagnosis is considered, often prompted only by treatment failure or an atypical clinical course. This diagnostic delay carries significant consequences, including malnutrition, irreversible mucosal damage, treatment toxicity and deferred access to targeted immunological therapy.

No standardised approach to endoscopic assessment or histopathological reporting in this population currently exists. The authors sought to address this gap through a collaborative Australian multicentre audit, with the aim of characterising the clinical and histopathological features of IEI/AEI-associated enteropathy across a broad spectrum of IEI/AEI diagnoses to contribute to the development of a clearer diagnostic and therapeutic framework.

## 2 Materials and methods

### 2.1 Literature review

PubMed was searched in April 2025 for English-language case reports and case series describing enteropathy associated with IEI using the following search string: (“primary immunodeficiency” OR “inborn errors of immunity” OR “common variable immunodeficiency” OR “CVID” OR “antibody deficiency” OR “immune dysregulation” OR “combined immunodeficiency” OR “hypogammaglobulinemia”) AND (“enteropathy” OR “gastrointestinal” OR “diarrhoea” OR “malabsorption” OR “colitis” OR “villous atrophy” OR “bowel disease”). No date restriction was applied. Reference lists of relevant articles were searched to capture additional reports. Studies were included if they reported non-infectious gastrointestinal disease in patients with a confirmed or probable IEI/AEI diagnosis and documented endoscopic or histopathological findings. Histopathological data were extracted and synthesised to identify the most frequently reported mucosal patterns, including IEL, villous atrophy, plasma cell depletion, apoptotic bodies, NLH, IBD-like changes, and GVHD-like changes, and to estimate the frequency with which each was observed across the published literature.

### 2.2 AT-SEA working group

In parallel, we established the Adult Tertiary Severe Enteropathy Audit (AT-SEA) working group, a collaborative network of Australian clinical immunologists and members of the Clinical Immunogenomics Research Consortium of Australasia (CIRCA). An expression-of-interest survey was distributed to 256 CIRCA members, which includes clinical immunologists, geneticists, specialist doctors-in-training and scientists, to identify clinicians managing patients with severe gastrointestinal disease in the context of IEI/AEI, and whose treating institution had CIRCA-established ethics approval for data collection. Eleven clinicians from 9 centres contributed de-identified patient data via a standardised survey (provided as **Supplementary Material**) covering demographics, immune diagnosis and International Union of Immunological Societies (IUIS) classification, genetic testing, gastrointestinal symptom onset, the clinician’s global assessment of severity based on symptoms and coexisting malabsorption, endoscopic and histopathological findings, nutritional complications, immunoglobulin replacement and dose escalation, and immunosuppressive and biologic treatment history.

#### 2.2.1 Inclusion criteria

Patients with a genetically or clinically confirmed IEI/AEI diagnosis and significant inflammatory gastrointestinal disease, defined by endoscopic histological abnormalities with any of the following: malabsorption, iron deficiency, protein-losing enteropathy, unintentional significant weight loss attributable to gastrointestinal disease, and/or persistent gastrointestinal symptoms of greater than three months duration. Included diagnoses encompassed IBD in the context of IEI/AEI, CVID-associated nodular lymphoid hyperplasia, coeliac or coeliac-like disease (responsive or unresponsive to gluten-free diet), and chronic or recurrent gastrointestinal infections in the setting of immune dysregulation. Patient recruitment occurred between August 2025 and June 2026.

#### 2.2.2 Exclusion criteria

Self-limited or short-duration gastrointestinal infections (less than eight weeks), very early onset IBD (VEO-IBD) diagnosed before six years of age, and gastrointestinal disease primarily attributable to Chronic granulomatous disease (CGD), X-linked inhibitor of apoptosis protein (XIAP) deficiency, or Immunodysregulation polyendocrinopathy enteropathy X-linked (IPEX) syndrome. CGD, XIAP deficiency, and IPEX syndrome were excluded as their gastrointestinal manifestations are mechanistically distinct and increasingly well-characterised, through NADPH oxidase-dependent granulomatous colitis, autoinflammatory colitis, and FOXP3-mediated autoimmune enteropathy respectively, and their inclusion would risk conflating pathophysiologically separate entities with the less well-characterised enteropathies that are the primary focus of this study. VEO-IBD was excluded on the same basis.

#### 2.2.3 Statistical analysis

This study was approved via CIRCA by the Sydney Local Health District RPAH Zone Human Research Ethics Committee and Research Governance Office, Royal Prince Alfred Hospital, Camperdown, NSW, Australia (Protocol X20-0177 & 2020/ETH0098). Informed consent was obtained from participants and the study was conducted in accordance with local regulations and with the approval of the institutional review boards of corresponding institutions. Data were fully de-identified prior to collation. Statistical analysis and data visualisation were performed using R (version 4.6.0; R Foundation for Statistical Computing, Vienna, Austria) with the ggplot2, dplyr, tidyr, readr, and dunn.test packages. Descriptive statistics were used to summarise the frequency of clinical, endoscopic, histopathological, and treatment-related variables across the cohort, with results presented as counts and percentages. Differences in histopathological pattern prevalence between primary antibody deficiency and non-primary antibody deficiency groups were assessed using Fisher’s exact test. The association between disease severity and number of histopathological patterns was evaluated using the Kruskal-Wallis rank sum test, with *post-hoc* pairwise comparisons performed using Dunn’s test with Bonferroni correction. A p value of less than 0.05 was considered statistically significant.

## 3 Results

### 3.1 Cohort demographics and clinical features

Twenty-eight patients with IEI/AEI-associated enteropathy were identified across the AT-SEA network, comprising 15 males and 13 females (**Table 1**). The median age at IEI/AEI diagnosis was 26 years (IQR 14–37) and the median age at onset of gastrointestinal symptoms suggestive of enteropathy was 28 years (IQR 15-44). The majority of patients carried a diagnosis of predominantly antibody deficiency (IUIS category 2, n=19, 68%), of whom 15 had CVID, with the remainder comprising diseases of immune dysregulation (category 3, n=5), combined immunodeficiencies with syndromic features (category 1, n=3), and one phenocopy of IEI/AEI (category 9). Genetic testing was performed in 20 of 28 patients (71%) with a probable or certain pathogenic variant identified in 14 of these patients. Specifically, of the 15 patients with a diagnosis of CVID, 9 underwent genetic testing with a pathogenic variant identified in only 3 (*TNFR*, *ADA2* and *NFKB1*). A positive family history of IEI/AEI-associated disease was present in 6 of 28 (21%) patients. Extra-intestinal manifestations were highly prevalent, present in 27 of 28 patients (96%), most commonly pulmonary (n=17, 61%), dermatological (n=15, 54%), and haematological involvement (n=14, 50%), with rheumatological features in 7 (25%) and malignancy in 5 (18%).

**Table 1:** Cohort characteristics (demographics).

Characteristic	n*	% of cohort
Demographics		
Total patients	28	
Female sex	13	46%
Median age	40 years	
Median age at IEI diagnosis, years (IQR)	26 years	
Median age at enteropathy onset, years (IQR)	28 years	
Positive family history of IEI/AEI	6	21%
Genetic testing performed	20	71%
Pathogenic or likely pathogenic variant identified	14	50
Ethnicity		
Caucasian/European	23	82%
East Asian	1	3%
South Asian	2	7%
Middle Eastern	2	7%
IUIS Classification Category		
Category 1 - Combined immunodeficiencies with associated or syndromic features	4	14%
Category 2 - Predominantly antibody deficiencies	18	64%
Category 3 - Diseases of immune dysregulation	4	14%
Category 7 - Phenocopies of IEI	2	7%
Specific Diagnosis (pathogenic gene)		
Common Variable Immunodeficiency (CVID)	18	64%
CTLA-4 haploinsufficiency *(CTLA4)*	3	10%
Good’s Syndrome	1	3%
Activated PI3K Delta Syndrome 1 (APDS1) *(PIK3CD)*	1	3%
X-linked SCID (post haemopoietic stem cell transplant) *(IL2RG)*	1	3%
PU.MA/PU.1 deficiency *(SPI1)*	1	3%
PTEN-associated immunodeficiency *(PTEN)*	1	3%
Other/unclassified	1	3%
Extra-intestinal Manifestations		
Any extra-intestinal manifestation	27	96%
Pulmonary	17	61%
Haematological	14	50%
Dermatological	15	54%
Rheumatological	7	25%
Endocrinological	6	21%
Neurological	4	14%
Renal	4	14%
Malignancy	5	18%

IQR, interquartile range; IUIS, International Union of Immunological Societies; CVID, common variable immunodeficiency; APDS, Activated PI3K Delta Syndrome; SCID, severe combined immunodeficiency; GI, gastrointestinal. *n refers to absolute number and does not apply to patient age, which is expressed in years.

Gastrointestinal disease severity was moderate or severe in 25 of 28 patients (89%), with 11 patients (39%) classified as severe and 14 (50%) as moderate (**Table 2**). Malabsorption was present in 20 of 28 patients (71%), unintentional significant weight loss in 18 (64%), iron deficiency in 13 (46%), and protein-losing enteropathy in 12 (43%). Enteral and/or parenteral nutritional support was required in 4 patients (14%). Immunoglobulin replacement was established in the majority of patients (22/28, 78%), with dose or frequency escalation required in 10 of 28 patients (36%), most commonly in the setting of protein-losing enteropathy (8/12, 67%) and severe disease (7/11, 64%). In 5 of 25 patients with available data, gastrointestinal symptoms preceded the IEI/AEI diagnosis, in two cases by over two decades, while in 11 patients the IEI/AEI diagnosis was established first with gastrointestinal symptoms developing a median of 5 years later. Enteropathy and IEI/AEI were diagnosed concurrently in 9 patients.

**Table 2:** Gastrointestinal disease severity.

Gastrointestinal disease severity		
Mild	3	11%
Moderate	14	50%
Severe	11	39%
Gastrointestinal features		
Malabsorption	20	71%
Iron deficiency	13	46%
Protein-losing enteropathy	12	43%
Unintentional significant weight loss	18	64%
Persistent symptoms >3 months	28	100%
Requirement for enteral nutrition	2	7%
Requirement for parenteral nutrition	2	7%
Endoscopic abnormalities (macroscopic)	16	57%
Histological abnormalities	26	93%

### 3.2 Histopathological patterns

A total of 48 biopsy reports were available across 28 patients, with pre-treatment results in all patients and post-treatment results in some. Our study demonstrated marked histological heterogeneity. To simplify this, findings were grouped into six categories based on the most frequent abnormalities reported: coeliac-like changes (including villous atrophy, increased IEL and crypt hyperplasia), reduced or absent plasma cells, nodular or diffuse lymphoid hyperplasia, IBD-like changes (including cryptitis, crypt architectural distortion, crypt abscesses), GVHD-like changes or increased apoptotic bodies, and intraepithelial neutrophilia. Of note, histological findings were only included when requiring specific comment by the reporting pathologist, including clear markers of histological injury (such as villous atrophy) or findings which were abnormal in quantitation or distribution (such as increased intraepithelial lymphocytes or absent plasma cells).

Across individual biopsy reports, increased IEL were identified in 22/48 (46%), villous atrophy in 22/48 (46%), IBD-like changes in 15/48 (31%), NLH in 16/48 (33%), reduced or absent plasma cells in 15/48 (31%), GVHD-like changes or apoptotic bodies in 11/48 (23%), crypt hyperplasia in 9/48 (19%), and intraepithelial neutrophilia in 8/48 (17%). These findings are illustrated in **Figure 1**.

**Figure 1:**
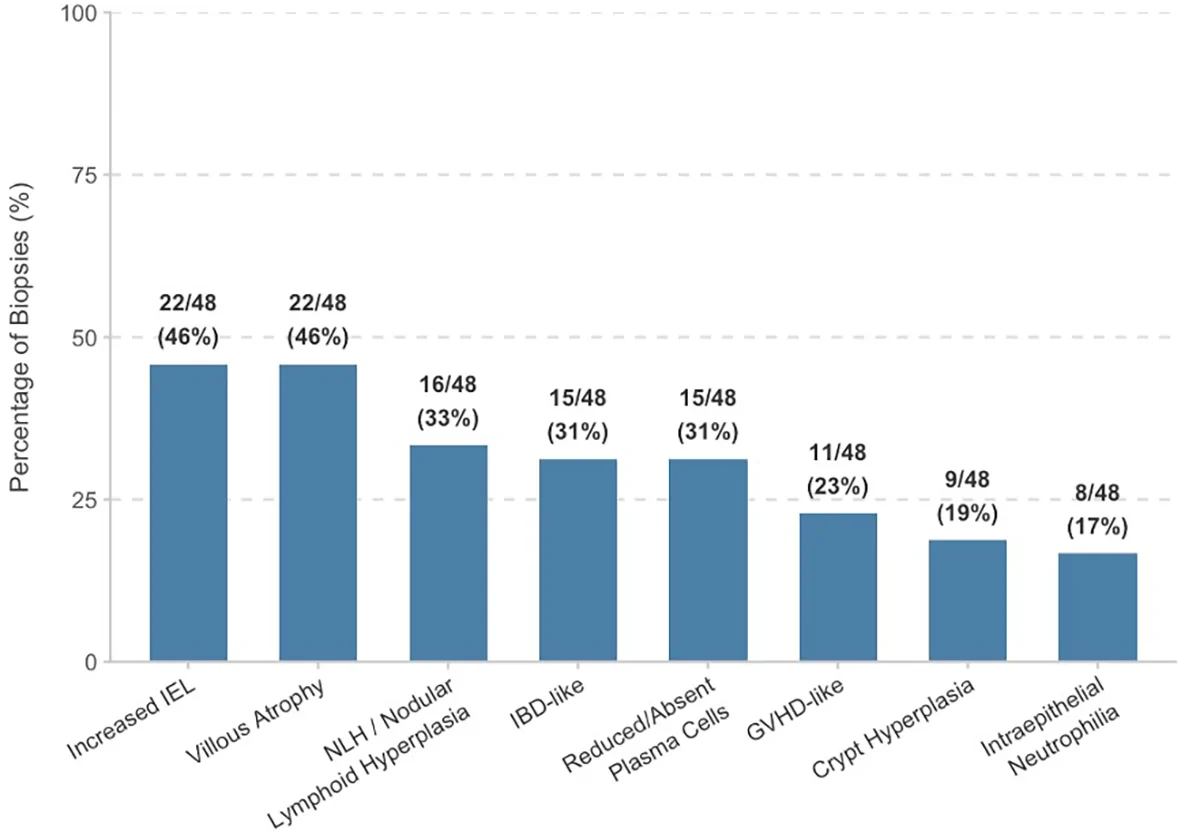
Frequency of histopathological patterns identified across individual small and/or large bowel biopsy reports in the AT-SEA cohort (n=48). Each bar represents the proportion of biopsy reports in which the specified histopathological pattern was identified. Data are reported at the biopsy level, incorporating all available upper and lower gastrointestinal endoscopic assessments across the cohort, including multiple biopsies from individual patients over time. Coeliac-like disease was defined as the presence of any of the following: increased intraepithelial lymphocytes, villous atrophy, or crypt hyperplasia. IBD-like changes include cryptitis, crypt abscess, crypt distortion, and granuloma formation. GVHD-like changes were defined by the presence of apoptotic bodies. NLH, nodular lymphoid hyperplasia; IEL, intraepithelial lymphocytes; GVHD, graft-versus-host disease; IBD, inflammatory bowel disease.

At the patient level, histological overlap was pronounced (**Figure 2**). A coeliac-like pattern was identified in 18 of 28 patients (64%), IBD-like changes in 12 (43%), NLH in 13 (46%), reduced or absent plasma cells in 11 (39%), GVHD-like changes in 7 (25%), and intraepithelial neutrophilia in 7 (25%). Importantly, 77% of patients demonstrated two or more concurrent histological patterns and 50% had three or more, with sequential biopsies demonstrating longitudinal evolution of patterns over time, some features resolving and new ones emerging. The co-occurrence of multiple patterns across patients is illustrated in **Figure 3**. Two patients did not demonstrate any histological changes on GI biopsy meeting our pre-defined categories and only demonstrated a non-specific chronic inflammatory cell infiltrate. Histopathological pattern frequency by diagnosis category is presented in **Table 3** (**Supplementary**).

**Figure 2:**
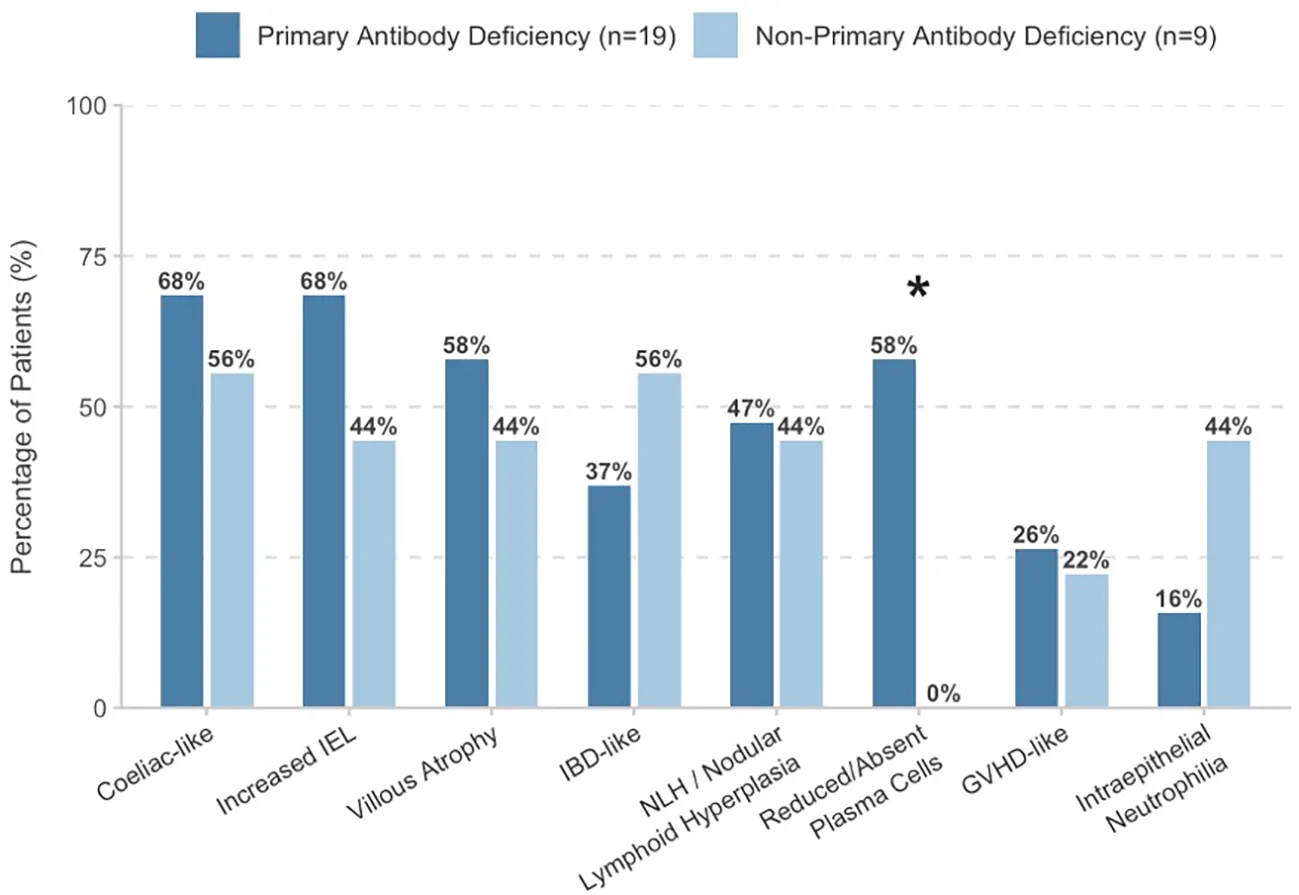
Histopathological patterns by immunological diagnosis category in the AT-SEA cohort (n=28). Bars represent the proportion of patients within each diagnosis category in whom the specified pattern was identified across all available biopsies. Primary Antibody Deficiency (IUIS category 2, n=19) includes predominantly CVID. Non-Primary Antibody Deficiency (n=9) includes combined immunodeficiency, immune dysregulation disorders, and phenocopies of IEI. *Reduced/absent plasma cells was the only pattern to differ significantly between groups (58% PAD vs 0% non-PAD; p=0.004, Fisher’s exact test); Intraepithelial neutrophilia and IBD-like changes were numerically more prevalent but not statistically significant in non-primary antibody deficiency patients (44% vs 16%, p=0.165 and 56% vs 37%, p=0.432 respectively).

**Figure 3:**
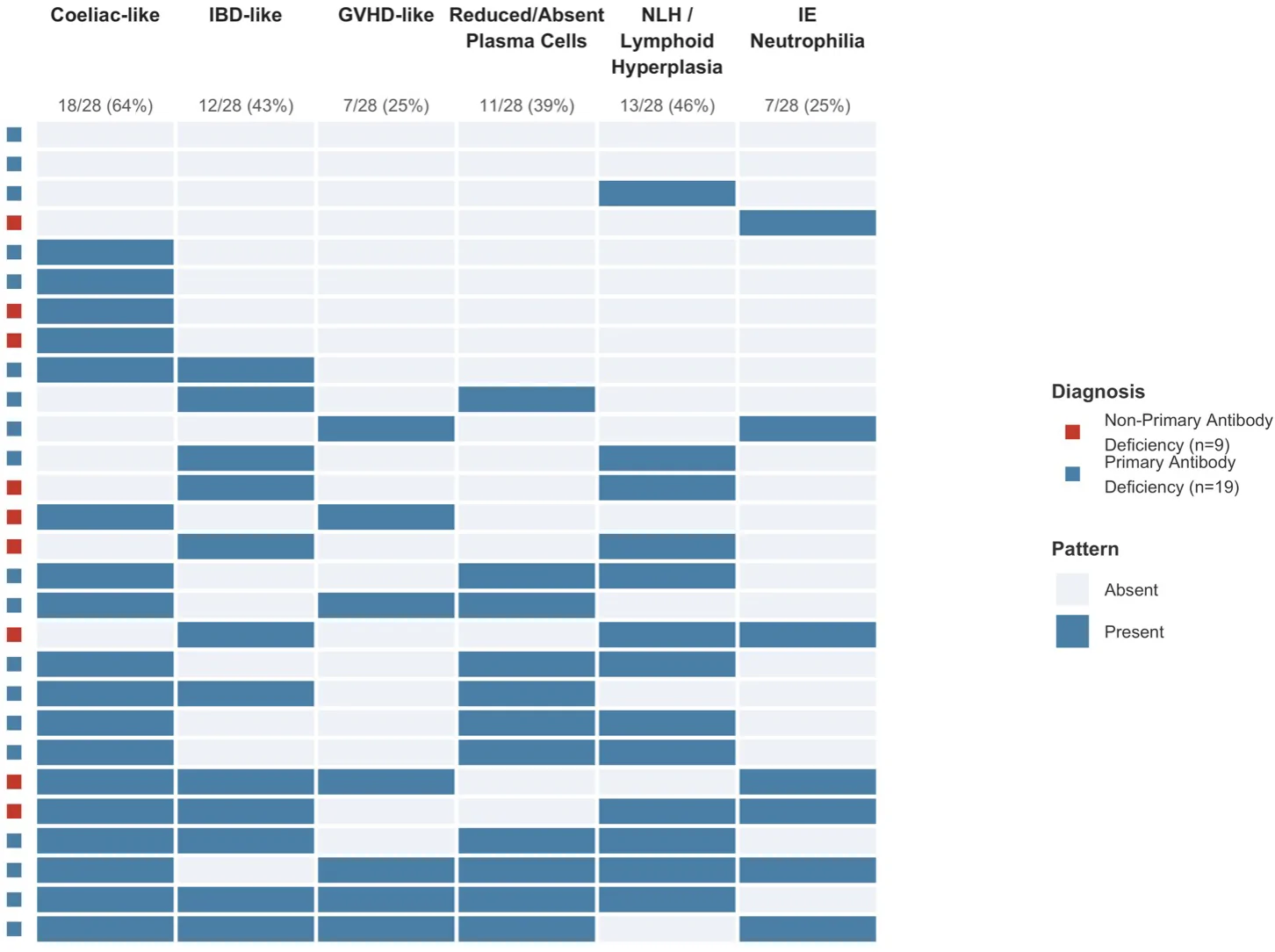
Co-occurrence of histopathological patterns across individual patients in the AT-SEA cohort (n=28). Each row represents one patient and each column one histopathological pattern category. Patients are ordered from most to least number of patterns identified (top to bottom).

**Table 3:** Histopathological pattern frequency by diagnosis category with Fisher’s exact test p values.

Histological pattern	Primary antibody deficiency (n=19)	Non-primary antibody deficiency (n=9)	Odds ratio	P Value
Coeliac-like	13 (68%)	5 (56%)	1.73	0.677
Increased IEL	13 (68%)	4 (44%)	2.71	0.409
Villous Atrophy	11 (58%)	4 (44%)	1.72	0.689
IBD-like changes	7 (37%)	5 (56%)	0.47	0.432
NLH/Lymphoid Hyperplasia	9 (47%)	4 (44%)	1.12	1.00
Reduced/Absent Plasma Cells	11 (58%)	0 (0%)	–	0.004
GVHD-like	5 (26%)	2 (22%)	1.25	1.000
Intraepithelial Neutrophilia	3 (16%)	4 (44%)	0.23	0.165

Reduced/absent plasma cells was the only pattern to differ significantly between groups. An odds ratio greater than 1 indicates the pattern is more prevalent in PAD.

Reduced or absent lamina propria plasma cells were identified in 15 of 48 biopsies (31%) and in 11 of 28 patients (39%) over their longitudinal disease course, all of whom carried a diagnosis of CVID. Plasma cell depletion was significantly more prevalent in primary antibody deficiency (PAD) compared to non-PAD patients (58% vs 0%, p=0.004, Fisher’s exact test). Across the 21 biopsy samples from CVID patients in whom plasma cell depletion was documented, reduced or absent plasma cells were identified in 3 gastric, 9 duodenal, 3 ileal, and 6 colonic samples, reinforcing the importance of sampling across all bowel segments.

### 3.3 Correlation with disease severity and malabsorption

There was a statistically significant difference in the number of histopathological patterns across clinician-assessed severity groups (Kruskal-Wallis chi-squared=6.34, df=2, p=0.042). Patients with severe disease had a median of 3 patterns per patient compared to 2 in moderate and 1 in mild disease (**Figure 4**). *Post-hoc* Dunn testing with Bonferroni correction identified a significant difference between mild and severe disease (p=0.018), while mild vs moderate (p=0.067) and moderate vs severe (p=0.557) did not reach significance, likely reflecting the small number of mild cases (n=3). Intraepithelial neutrophilia showed the most pronounced severity gradient, present in 36% of severe, 14% of moderate, and 0% of mild cases. The coeliac-like pattern was present in 82% of severe, 64% of moderate, and 33% of mild patients. Notably, villous atrophy did not reliably predict the presence of malabsorption: villous atrophy was present in 73% of patients both with and without nutritional compromise (p=1.0, Fisher’s exact test).

**Figure 4:**
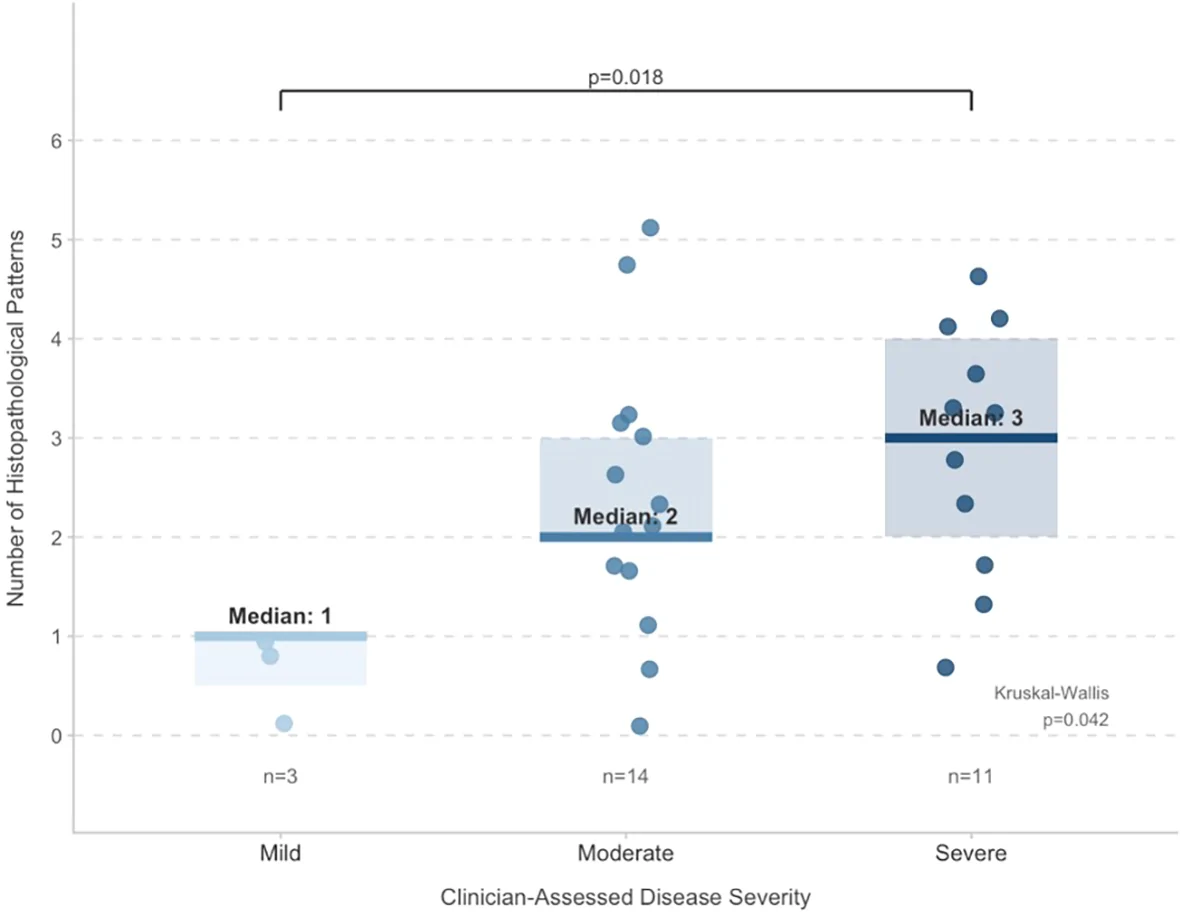
Number of histopathological patterns identified per patient based on disease severity in the AT-SEA cohort. Each dot represents one patient. The shaded box represents the interquartile range (IQR) and the horizontal line the median number of patterns within each severity group. There was a statistically significant difference in the number of histopathological patterns across severity groups (Kruskal-Wallis chi-squared=6.34, df=2, p=0.042). *Post-hoc* Dunn testing with Bonferroni correction identified a significant difference between mild and severe disease (p=0.018), while mild vs moderate (p=0.067) and moderate vs severe (p=0.557) comparisons did not reach significance. Disease severity was assigned by the treating clinician based on clinical, biochemical, and endoscopic assessment.

### 3.4 Treatment

Corticosteroids were used in the majority of patients, with oral budesonide in 11/28 (39%) and oral prednisolone in 10/28 (36%). Sixty-five percent of patients required systemic immunosuppression, with antimetabolites (azathioprine, mycophenolate mofetil, or 6-mercaptopurine) used in 11/28 (39%). Biologic agents were used in 8/28 (29%), including vedolizumab, ustekinumab, infliximab, adalimumab, rituximab, and abatacept. Vedolizumab was used in 4 patients (2 CVID, 1 Good’s syndrome, 1 immune dysregulation syndrome) without significant improvement, however one patient with CVID showed partial response to ustekinumab subsequently. One patient with PU.1 deficiency showed improvement on infliximab. One patient with immune dysregulation syndrome with positive Glutamic Acid Decarboxylase (GAD) antibody showed improvement on tacrolimus and abatacept together. All patients requiring immunomodulatory treatment except one required a range of 2–6 agents over their disease course. Treatment response was based upon the clinician’s assessment. Due to the heterogeneity of histopathological findings, the small numbers of each specific IEI/AEI diagnosis and the variable responses to different treatments, details of individual patients’ histological changes following treatment have not been presented.

Mammalian target of rapamycin (mTOR) inhibition with sirolimus was used in 10/28 (36%) patients and was predominantly prescribed in non-primary antibody deficiency disorders (7/10). Sirolimus was identified as the most effective agent in patients with Cytotoxic T-Lymphocyte Associated Protein-4 (CTLA-4) haploinsufficiency, Activated Phosphoinositide 3-Kinase Delta Syndrome (APDS), Phosphatase and TENsin homolog (PTEN)-associated immunodeficiency, and combined immunodeficiency; in one patient with PTEN-associated immunodeficiency, a combination of sirolimus and ustekinumab achieved good disease control. Among primary antibody deficiency patients, responses were more heterogeneous: antimetabolites achieved sustained remission in several CVID patients, one patient responded to mesalazine alongside commencement of subcutaneous immunoglobulin, and one achieved remission following rituximab administered primarily for a haematological indication. Janus Kinase (JAK) inhibitors were used in 2 patients (7%), with moderate improvement in one patient with CVID on upadacitinib (however limited by acne as a side effect), and no effect in another patient with immune dysregulation syndrome on tofacitinib.

## 4 Discussion

This study represents the first Australasian cohort contribution to the characterisation of IEI/AEI-associated enteropathy. It highlights that IEI/AEI-associated enteropathy is characterised by overlapping and evolving histological patterns rather than discrete subtypes, and that the co-occurrence of multiple patterns should prompt consideration of an underlying IEI/AEI. The presence of several abnormalities, which in isolation might be considered minor, may have greater significance when identified together in this context. The significant correlation between histological complexity and clinician-assessed disease severity, the strong enrichment of plasma cell depletion in primary antibody deficiency disorders, and the divergent treatment responses between different IEI/AEI-subtypes, collectively support a precision immunology approach to diagnosis and management.

The histopathological findings in our study are broadly consistent with data from published series describing coeliac-like, IBD-like, GVHD-like and other patterns coexisting within the same cohort, and sometimes within the same patient over time, as summarised in **Table 4** (8, 17–20). The presence of two or more concurrent or evolving histological patterns in a patient with chronic gastrointestinal disease should prompt consideration of an underlying IEI/AEI. Notably, compared to other case series/reports listed in **Table 4**, the AT-SEA cohort is the only one in which all histological pattern categories are represented. This likely represents a combination of factors: deliberate recruitment of patients with moderate to severe enteropathy through a specialist network, broader diagnostic inclusivity beyond CVID to include immune dysregulation disorders and reporting bias, with our authors’ conscious effort to include these reported histological changes which may have been present but under-reported in other studies. The predominantly Caucasian demographic (82%) reflects the known epidemiology of CVID in Australia and limits the generalizability to other populations.

**Table 4:** Frequency of histopathological patterns in IEI/AEI-associated enteropathy across published case series and the AT-SEA cohort.

Study	Disease location	Total number of patients assessed	IEL	Apoptotic bodies	Villous atrophy	Reduced/absent plasma cells	NLH/diffuse follicular lymphoid hyperplasia	IBD-like changes*	Intraepithelial neutrophilia
A (3)		19	++*	+	+	–	+	+++	–
B (24)		53	+++	-	-	+++	++	-	-
C (44)		3	++++	–	++++	+++	++	++	–
D (12)	Small bowel	19	+++	+	+++	+++	++	-	+
	Colon	16	++	++	-	+++	++++	++	++++
E (45)	Small bowel	41	+	-	+	++++	++	+	-
	Colon	29	+	-	-	++++	++	++	-
F (34)		1	-	-	-	++++	++++	++++	-
G (46)		7	++	++	+	+++	++	++	–
H (47)	Small bowel	8	+	-	++	-	-	-	-
	Colon	10	+++	-	-	-	-	+	-
I (16)		7	++++	+	++++	–	–	–	–
J (8)	Small bowel	41	+++	+	+++	++++	++	-	-
	Colon	35	++	+	-	-	++	+	-
K (48)	Small bowel	8	++++	+	++++	-	+	-	+
	Colon	8	+++	+	-	-	+++	+	++
L (49)	Small bowel	2	++++	-	++++	++++	-	++++	-
M (50)	Small bowel	32	–	–	++	–	+	–	–
N (51)	Small bowel	11	++	+++	++	-	+	+	-
	Colon	5	++++	+++	-	-	-	++	+
O (52)		5	+	–	+	–	–	+++	–
P (53)		1	-	+++	-	++++	-	-	-
Q (54)		1	–	–	–	++++	++++	++++	–
R (55)	Small bowel	1	-	-	++++	-	-	-	-
	Colon	4	++++	-	-	-	-	+	-
S (11)	Small bowel	21	+++	-	++++	++++	+++		-
	Colon	8	-	-	-	+++	-		++
T (56)	Small bowel	34	+++	-	++	++	+		+
	Large bowel	34	-	-	-	-	+		-
U (57)		3	–	–	–	–	–		–
V (58)		1	-	-	-	-	-		++++
AT-SEA Cohort	Small bowel and colon	28	**+++**	**+**	**+++**	**++**	**++**	**++**	**+**

Each row represents a published case series or cohort study reporting gastrointestinal histopathology in patients with inborn errors of immunity (IEI) or acquired errors of immunity (AEI). Where studies reported findings separately by bowel segment, small bowel and colonic findings are presented in separate rows. The frequency of each histopathological pattern within each study is represented using a semi-quantitative grading system based on the proportion of patients in whom the feature was identified: - (0%), + (1–30%), ++ (31–50%), +++ (51–80%), ++++ (81–100%). The AT-SEA cohort row (highlighted) represents findings from the current study (n=26); actual proportions are shown in parentheses alongside the corresponding grade to facilitate direct comparison with published data. IBD-like changes include cryptitis, crypt hyperplasia, crypt abscess, crypt distortion, and granuloma formation. Studies are identified by letter with corresponding references.

IEI/AEI-associated enteropathy shares many histological features with classical gastrointestinal diseases, making distinction based on histology alone challenging, yet several patterns of similarity and divergence are clinically informative. While a coeliac-like pattern was the most frequent finding in the AT-SEA cohort, present in 57% of patients across time, 13 of 16 patients with coeliac-like histology also displayed one or more features atypical for true CD, including apoptotic bodies, NLH, reduced or absent plasma cells, IBD-like changes, and intraepithelial neutrophilia. Additional subtle distinguishing features from true CD include a relatively lower IEL count, acute duodenitis with neutrophilic infiltration, presence of granulomata and subepithelial band thickening (21, 22). However, these features are variable and inconsistent, and the most clinically useful discriminator remains a failure to respond to gluten-free diet (8, 9, 12). Similarly, IBD-like changes were present in 39% of patients in our cohort, however 6/11 of these patients had concurrent small bowel coeliac-like involvement - a distribution atypical of true IBD (20). Key distinguishing features of Crohn’s Disease include the presence of non-caseating granulomas and greater tendency to cause complications such as strictures and fistulae (5, 9).

Reduced or absent lamina propria plasma cells are widely cited as one of the most discriminating features of CVID-associated enteropathy, present in 60–80% of biopsies from patients with predominantly antibody deficiency diseases in published series (9, 12, 17, 23–25). In the AT-SEA cohort, plasma cell depletion was significantly more prevalent in primary antibody deficiency (PAD) compared to non-PAD patients (58% vs 0%, p=0.004, Fisher’s exact test), and was the only histological pattern to differ significantly between diagnostic subgroups. Notably, other IEIs such as Good’s Syndrome, APDS, PU.1 deficiency and CTLA4 haploinsufficiency did not share this finding. The presence of plasma cells on intestinal biopsy therefore does not exclude IEI/AEI-associated enteropathy, especially in the conditions listed above or when the underlying IEI/AEI is undiagnosed, and reliance on their absence as a diagnostic criterion risks missing a substantial proportion of patients, particularly those with immune dysregulation rather than pure antibody deficiency. Like other case studies, the CVID patients in the AT-SEA cohort demonstrated heterogenous distribution of reduced plasma cells across the small and large bowel (17, 24), highlighting the importance of obtaining multiple GI tract biopsies for adequate assessment. Although intraepithelial neutrophilia and IBD-like changes were numerically more prevalent in non-PAD patients 44% vs 16%, p=0.165 and 56% vs 37%, p=0.432 respectively), these differences did not reach statistical significance in this small cohort and may warrant evaluation in larger studies as potential discriminating features between diagnostic subgroups (See **Supplementary Table 1**).

This is the first study to demonstrate a statistically significant correlation between clinician-assessed disease severity and histopathological complexity in IEI/AEI-associated enteropathy (Kruskal-Wallis p=0.042). Patients with severe disease had a median of three patterns per patient compared to one or less in mild disease (p=0.018), suggesting that increasing histological burden, rather than any single pattern, may be the most meaningful correlate of clinical severity. Intraepithelial neutrophilia showed the most pronounced gradient, present in 36% of severe and none of the mild cases, although this did not reach significance. This is consistent with its role as a marker of acute innate immune-driven mucosal injury, a mechanism well described in intestinal inflammation, where excessive neutrophil transepithelial migration causes epithelial barrier disruption and amplifies the inflammatory cascade (26), and warrants active exclusion of superimposed infection at the time of biopsy. Notably, villous atrophy did not reliably predict malabsorption, present in 73% of patients both with and without nutritional compromise, suggesting that malabsorption in IEI/AEI-associated enteropathy reflects mechanisms beyond architectural mucosal damage alone, including microbial dysbiosis and impaired secretory function (16, 27, 28). This suggests that individual histological features should not be used in isolation to assess nutritional risk, and that all patients with IEI/AEI-associated enteropathy warrant close nutritional monitoring regardless of histological pattern.

There is currently no consensus on the indication or regime of treatment for IEI/AEI-associated enteropathy, however symptomatic patients with suggestive histology should be considered for immunosuppressive treatment after infectious causes are excluded (29). Treatment data from our cohort highlights both the limitations of the conventional IBD-based stepwise approach and the emerging role of molecularly targeted agents (6, 30–34). While vedolizumab and ustekinumab have shown promise in some cases of steroid-refractory CVID-enteropathy (35–37), responses were variable in our cohort with 3 out of 4 patients experiencing treatment failure, although patient numbers were too low to draw significant conclusions regarding efficacy. The predominant and successful use of sirolimus in non-primary antibody deficiency patients reflects the mechanistic rationale for mTOR inhibition in T-cell dysregulation disorders and is consistent with a growing body of evidence supporting its use for enteropathy associated with Lipopolysaccharide-responsive and Beige-like Anchor Protein (LRBA) deficiency, CTLA-4 haploinsufficiency, APDS and IPEX (38–42). These observations reinforce that biologic selection must be guided by the underlying immune defect and genetic findings rather than extrapolated from IBD treatment algorithms.

Notably, although we excluded very early onset presentations, the late median age at diagnosis of IEI/AEI (26) highlights the importance of awareness IEI/AEIs presenting in adulthood. Enteropathy can present early in disease course and even precede the IEI/AEI diagnosis, as observed in 5/28 of patients in this cohort. Furthermore, a recent large-scale genome sequencing study found that approximately 1% of patients with well-phenotyped common disease diagnoses, including IBD, harboured an underlying monogenic disorder, most commonly related to immunodeficiency, resulting in delayed and inappropriate treatment (43). These findings collectively emphasise the importance of maintaining a low threshold for immunology referral in adult patients with treatment-refractory or atypical gastrointestinal disease, even in the absence of a prior immunological diagnosis or childhood history of recurrent infections.

This study has several limitations inherent to its retrospective multicentre design, including incomplete longitudinal data for some patients, particularly those transitioning from paediatric to adult services where historical records were fragmented, and reliance on clinician-completed surveys that did not capture standardised treatment sequencing or response data. Detailed data regarding measures of response to treatment in the long term and final outcomes were not collected, and due to small numbers of patients with each underlying IEI/AEI, this study was not powered to assess response to treatment. Heterogeneity in endoscopic practice across contributing centres, including variation in whether upper, lower, or combined endoscopy was performed and the number and distribution of biopsies obtained, together with non-standardised pathology reporting, may have resulted in inconsistent documentation of subtle histological features. The modest cohort size limits the interpretation of statistical significance of results. Australia has a relatively small population spread over a large geographical area, with clinicians treating patients with IEI far apart in different states and health districts, which adds a challenge to collaborative research into rare diseases including IEI. The establishment of CIRCA has been invaluable in bringing together clinicians with IEI expertise and enabling ethical approval for multicentre collaborations, however not all clinical immunologists treating IEI work in centres with CIRCA-facilitated ethics approval, which limited recruitment. Further work to expand collaborations to include more clinicians and thus recruit more patients into this type of study is needed.

Several important practice implications emerge from these findings. Given the patchy and pan-enteric distribution of mucosal disease demonstrated in this cohort, adequate histopathological assessment requires multiple biopsies from both upper and lower gastrointestinal segments, including from macroscopically normal mucosa, as reliance on a single site risks missing diagnostically significant findings. Reinforcing this, 57% of patients within our cohort had a normal macroscopic appearance at endoscopy, while 93% had histological abnormalities. Furthermore, given the longitudinal evolution of histopathological patterns observed in patients with sequential biopsies, clinicians should consider re-evaluating disease with repeat endoscopy over time, particularly at points of clinical change or treatment failure, as the concomitant presence of features spanning multiple disease categories may only become apparent across serial assessments. Consequently, clinical diagnosis and the evaluation of treatment response necessitate longitudinal (i) clinical, (ii) endoscopic and (iii) histopathological assessments to improve clinical outcomes. These findings provide a foundation for larger prospective multicentre registries incorporating central pathology review, standardised biopsy protocols, and uniform data collection, which will be essential to correlating histopathological patterns with immune phenotype and genetic diagnosis, refining diagnostic criteria, and informing precision-based therapeutic strategies in this under-recognised condition.

## 5 Conclusion

IEI/AEI-associated enteropathy is a heterogeneous and under-recognised condition. The AT-SEA cohort contributes to the growing literature describing the histopathological heterogeneity associated with the condition. We identified that coexistence of multiple hallmark features of classical GI diseases should alert the physician to the possibility of underlying IEI/AEI, and that increasing histological complexity may correlate with disease severity. Prospective multicentre studies with standardised endoscopic protocols, central pathology review, and molecular diagnostic integration will be essential to refining diagnostic criteria and informing precision-based therapeutic strategies in this under-recognised and clinically challenging condition.

## Data Availability

The datasets for this article are not publicly available due to concerns regarding participant/patient anonymity. Requests to access the datasets should be directed to the corresponding author.
