# Supplementary material for: Enteropathy in inborn and acquired errors of immunity: histological patterns and clinical correlates
Source: Front Immunol. 2026 Oct 15;17:1934715. doi: 10.3389/fimmu.2026.1934715 (PMC13716641; doi:10.3389/fimmu.2026.1934715)
Supplement: Supplementary file 1 [file Supplementaryfile1.docx]

**Supplementary Data Sheet 1:** Adult Tertiary Severe Enteropathy Aduit (AT-SEA) Working Group Survey. Standardised survey distributed to participating clinicians for data collection.

## 1. Demographics

- Initials (*e.g. MT*):
- Date of Birth:
- Sex:
- Ethnicity/Country of Birth:

## 2. PID diagnosis

- Condition and/or IUIS phenotypical classification category (please tick relevant option/s):

☐Combined immunodeficiencies with associated or syndromic features

☐Predominantly antibody deficiencies

☐Diseases of immune dysregulation

☐Congenital defects of phagocyte number or function

☐Defects in intrinsic and innate immunity

☐Autoinflammatory disorders

☐Complement deficiencies

☐Bone marrow failure

☐Phenocopies of inborn errors of immunity

- Please state the specific diagnosis:
- Genetic testing:

☐Yes (please specify the gene mutation identified):

☐No

- Age at diagnosis:
- Manifestations
  - Pulmonary

☐Yes (please specify):

☐No

- Endocrine

☐Yes (please specify):

☐No

- Haematological

☐Yes (please specify):

☐No

- Rheumatological

☐Yes (please specify):

☐No

- Dermatological

☐Yes (please specify):

☐No

- Neurological

☐Yes (please specify):

☐No

- Renal

☐Yes (please specify):

☐No

- Malignancy

☐Yes (please specify):

☐No

- Other (please specify):
- Family history of PID or immune-mediated gastrointestinal disease:

☐Yes (please specify):

☐No

## 3. Gastrointestinal disease

- Does this patient meet any of the following criteria for moderate to severe enteropathy associated with PID? (Please tick all relevant options)

☐Gross endoscopic abnormalities

☐Histologic abnormalities

☐Malabsorption

☐Iron deficiency

☐Protein loss

☐Unintentional significant weight loss related to GI disease

☐Persistent symptoms of duration > 3 months

**NOTE**

*DOES include: a diagnosis of “Inflammatory Bowel Disease,” Nodular Lymphoid Hyperplasia associated with CVID, Coeliac disease or Coeliac-like changes (e.g. villous atrophy) either responsive or not responsive to gluten-free diet*

*DOES NOT include: self-limited or short (<8 weeks) GI tract infections, VEO-IBD (diagnosed < age 6), GI disease related to CGD, XIAP and IPEX*

- Age at onset of symptoms or diagnosis of enteropathy:
- Severity of gastrointestinal symptoms:

☐Mild

☐Moderate

☐Severe

- Other gastrointestinal manifestations:

☐Nodular hyperplasia of the liver

☐Autoimmune liver disease

☐Hepatic steatosis

☐Chronic liver dysfunction of other/unknown cause

☐Non-cirrhotic portal hypertension

☐Autoimmune gastritis

☐Other (please specify):

- Complications of enteropathy (please tick relevant option/s)

☐Malabsorption - defined as any of the following micronutrient deficiencies (please tick all relevant options):

☐Iron

☐B12

☐Folate

☐Protein

☐Fat soluble vitamins (ADEK)

☐Weight loss

☐Failure to thrive

☐Severe electrolyte imbalance (symptomatic or requiring intravenous replacement)

☐Venous thromboembolism

- Treatment outline (please tick all options that have been used in the past or are currently in use):

☐Oral IVIG

☐Oral budesonide

☐Oral prednisolone

☐Antimicrobials

☐Systemic immunosuppression (please tick all relevant options and specify the name and duration of use for each if possible):

☐Antimetabolites (e.g. AZA, 6-MP, MTX):

☐Biologic agents (e.g. anti-TNF agents, CTLA-4 agonists, IL1/IL6 blockers, anti-IL12/23 agents):

☐mTOR inhibitors:

☐JAK inhibitors:

- Of all the above options, which treatment/treatment combinations were the most effective for your patient?
- Use of intravenous immunoglobulin (IVIG) vs subcutaneous immunoglobulin (SCIG) (please tick relevant option/s):

☐IVIG

☐SCIG

☐Both

☐None

- Need for increased dose or frequency of immunoglobulin administration to account for protein loss from enteropathy:

☐Yes

☐No

- Need for enteral or parenteral feeding (please tick relevant option/s):

☐Enteral nutrition (e.g. via NGT/NJT/PEG)

☐Parenteral nutrition (e.g. TPN)

☐Both

☐None

## 4. Faecal Testing

Please provide the following faecal testing results from the time of diagnosis (or as close to this time as possible) if available:

- Stool MCS, PCR, OCP
- Faecal calprotectin
- Faecal elastase
- Faecal A1AT

## 5. Endoscopy findings

Please attach a copy of the **deidentified** endoscopy report and biopsy results from the procedure closest to the time of diagnosis of enteropathy, AND any subsequent endoscopy/biopsy reports if available
